# Human umbilical cord-derived mesenchymal stem cells (hUC-MSCs) alleviate excessive autophagy of ovarian granular cells through VEGFA/PI3K/AKT/mTOR pathway in premature ovarian failure rat model

**DOI:** 10.1186/s13048-023-01278-z

**Published:** 2023-09-30

**Authors:** Wenjie Dai, Hong Yang, Bo Xu, Tiantian He, Ling Liu, Xiaoqian Ma, Jiaxue Ma, Guoqin Yang, Rui Si, Xiuying Pei, Xing Du, Xufeng Fu

**Affiliations:** Key Laboratory of Fertility Preservation and Maintenance of Ministry of Education, School of Basic Medical Sciences, Ningxia Medical University, Yinchuan, 750004 China

**Keywords:** Human umbilical cord-derived mesenchymal stem cells (hUC-MSCs), Premature ovarian failure, Ovarian granulosa cells, Excessive autophagy, PI3K/AKT/mTOR

## Abstract

**Background:**

Premature ovarian failure (POF) is one of the leading causes of female infertility and is accompanied by abnormal endocrine, seriously affecting female quality of life. Previous studies have demonstrated that mesenchymal stem cells (MSCs) transplantation is a promising therapeutic strategy for POF. However, the mechanism remains obscure. This study aims to investigate the therapeutic effect of human umbilical cord-derived mesenchymal stem cells (hUC-MSCs) on ovarian function in the POF rat model and explore the underlying mechanisms.

**Methods:**

The ovarian function was evaluated by ovarian morphology, histology, estrous cycle, hormone levels (AMH, E2, FSH, and LH), and fertility ability to investigate the effect of hUC-MSCs on the POF rats model. The cytokines levels were assayed in serum using protein array to explore the mechanisms of hUC-MSCs therapy for POF. The excessive autophagy levels were evaluated using a co-culture system of 3D MSCs spheroids with human ovarian granulosa cell line (KGN) or primary ovarian granulosa cells (GCs) to understand the paracrine effect of hUC-MSCs on GCs. The related proteins expression of autophagy and PI3K/AKT/mTOR pathway was detected using Western Blotting and/or in various inhibitors supplement to further demonstrate that vascular endothelial growth factor A (VEGFA) secreted by hUC-MSCs can alleviate excessive autophagy of ovarian GCs via PI3K/AKT/mTOR signaling pathway. The ovarian culture model in vitro was applied to confirm the mechanism.

**Results:**

The ovarian function of POF and the excessive autophagy of ovarian GCs were restored after hUC-MSCs transplantation. The protein array result demonstrated that VEGF and PI3K/AKT might improve ovarian function. in vitro experiments demonstrated that VEGFA secreted by hUC-MSCs could decrease oxidative stress and inhibit excessive autophagy of ovarian GCs via PI3K/AKT/mTOR pathway. The ovarian culture model results confirmed this mechanism in vitro.

**Conclusion:**

The hUC-MSCs can alleviate excessive autophagy of ovarian GCs via paracrine VEGFA and regulate the PI3K/AKT/mTOR signaling pathway, thereby improving the ovarian function of POF.

**Supplementary Information:**

The online version contains supplementary material available at 10.1186/s13048-023-01278-z.

## Background

The primordial follicles determine the number of oocytes at the birth of females. This ovarian reserve would progressively dwindle until left with only a thousand primordial follicles, determining a woman enters menopause around the age of 50 [1, 2]. Female fertility declines with chronological age, and ovarian dysfunction is frequently an important sign of human female aging [3]. The increase in atretic follicles and decreased oocyte quality ultimately result in the cessation of ovarian function, amenorrhea, and infertility during normal physiological aging [4]. Regarding pathological conditions, premature ovarian failure (POF) is a common reproductive disorder characterized by menstrual disturbances and deficient mature follicles accompanied by decreased estrogen levels and elevated gonadotropin levels before age 40 [5, 6]. Recently, POF incidence has increased, affecting approximately 1% of women under 40 and even 0.1% of women under 30 [7]. Additionally, POF is associated with the risk of other diseases, such as cardiovascular disease, osteoporosis, and sexual dysfunction, which can seriously affect a woman’s quality of life [8, 9]. However, current POF therapeutic schedules still have limitations in physiology or pathology, such as hormone replacement therapy, which is an ineffective solution to the infertility problem [10]. Therefore, exploring effective strategies to improve ovarian function may benefit POF patients’ physical, mental, and reproductive health.

In recent decades, mesenchymal stem cells (MSCs) therapy has been considered a promising measure in treating several human diseases and applications of regenerative medicine due to its self-renewal and differentiation abilities, paracrine cytokines, and exosomes, which can rescue injured tissues, including POF [11]. Numerous studies have verified that administering MSCs from various sources (bone marrow, adipose tissue, and menstrual blood) can ameliorate ovarian function and promote fertility in the POF model [12–14]. However, these MSCs sources involve invasive procedures or have a low magnitude of the original stem cells. The umbilical cord is a medical waste of perinatal tissue. The procedure for obtaining human umbilical cord-derived mesenchymal stem cells (hUC-MSCs) is noninvasive and has no ethical debate. Additionally, hUC-MSCs derived from young perinatal tissue demonstrated less virus infection, a higher proliferation rate, and fewer ethical concerns than MSCs derived from autologous sources [11]. Therefore, hUC-MSCs are considered an ideal cell type for treating tissue damage and have recently emerged as a promising clinical application candidate. The hUC-MSCs have demonstrated excellent properties in improving ovarian function and fertility in animal models of POF [11]. In a clinical trial study, hUC-MSCs have been applied to treat POF patients. The result demonstrated that hUC-MSCs rescued overall ovarian function, elevated estradiol concentrations, improved follicular development, and increased antral follicles, achieving clinical pregnancy [15]. These studies implied that hUC-MSCs therapy is a promising application strategy in treating female infertility for POF. Although hUC-MSCs have demonstrated satisfactory effects for ovarian function in animal models and clinical trials of POF, the mechanisms underlying their restorative effects remain unknown.

Autophagy is a self-renewal process for clearing dysfunctional proteins and organelles in eukaryotic cells. However, the excessive autophagy activation would induce the essential component digestion for cell survival, leading to self-destruction in an oxidative injury condition [16, 17]. Granulosa cells (GCs) play an important role in follicular development by secreting estradiol and several essential factors for oocyte maturation [18]. Recent studies suggested that oxidative stress-induced autophagy of GCs is crucial for follicular atresia [19]. Meanwhile, reducing excessive autophagy of GCs can improve POF ovarian function [20–23]. Thus, elucidating the preventive mechanisms against oxidative stress-triggered GCs’ excessive autophagy and identifying an effective antioxidant may provide plausible strategies for POF patients caused by aberrant follicular atresia. Numerous studies have demonstrated that MSCs have an antioxidative effect dependent on their secreted cytokines and exosomes [24–26]. Moreover, MSCs can reduce the oxidative stress level of GCs in MSCs improving ovarian function [27, 28]. Therefore, we proposed a hypothesis that hUC-MSCs restore the ovarian function of POF by reducing excessive autophagy of GCs.

This study aims to transplant hUC-MSCs into a cyclophosphamide-induced POF rat model by intravenous tail injection to evaluate the restorative effect on ovarian function. The results may indicate the important role of vascular endothelial growth factor A(VEGFA) in hUC-MSCs decreasing excessive autophagy of GCs. Then, we confirm the effects of hUC-MSCs and VEGFA inhibiting autophagy of GCs in vitro and explore the underlying mechanism further. The results revealed that hUC-MSCs and VEGFA reduced excessive autophagy of GCs through activation of the PI3K/AKT/mTOR pathway. Moreover, improving POF through inhibiting excessive autophagy of GCs would provide a new strategy for POF therapy.

## Materials and methods

### hUC-MSC preparation and culture

The hUC-MSCs were established and identified in our previous study [29]. This study was approved by the Institutional Ethics Committee of Ningxia Medical University. Briefly, hUC-MSCs were isolated from umbilical cord tissue by dividing it into equal-sized pieces and cultured with low-glucose Dulbecco’s modified Eagle’s Medium (DMEM, Gibco, USA) supplemented with 10% fetal bovine serum (FBS, Gibco, USA), and 1% penicillin-streptomycin (Gibco, USA) in a humidified atmosphere at 37 °C with 5% CO_2_. After one week, many fibroblast-like cells appeared, and the tissue fragments were removed. The primary cells were subcultured with 0.25% trypsin (Gibco, USA), suspended in medium, and subcultured in new plastic Petri dishes. The morphology, surface markers, and differentiation potential of MSCs were assessed in the third passage [29].

### PKH26-labeled hUC-MSCs tracking

The hUC-MSCs were labeled with PKH26 Red Fluorescent Cell Linker Mini Kit (Sigma, MINI26-1KT) according to its instruction. Briefly, hUC-MSCs were washed with serum-free medium and centrifuged at 400 *g* for 5 min, and the supernatant was discarded, then, 1 mL diluent C was added and gently mixed. The preparation of the dye is to mix 4 µL of PKH26 dye in 1 mL diluent C. Then, cell suspension and staining solution were mixed with the same volume and cultured in an incubator for 3 min, and 2mL FBS was added to terminate the reaction. Before using, cells were washed with the medium and centrifuged at 400 *g* for 5 min. The frozen section technique and immunofluorescence were performed to determine the location of the transplanted hUC-MSCs and their fate in the ovary. Three rats in each group were randomly selected to kill at 2, 7, and 14 days after PKH26-labelled hUC-MSCs transplantation. The ovary was collected, immersed in 20% sucrose solution for 2 h, and embedded with a frozen section embedding agent. Then, the ovaries were serially frozen and sectioned into 5 μm. All sections were visualized using fluorescence microscopy (Olympus, Japan).

### Animal experiments

Forty-eight five-week-old inbred female Sprague-Dawley (SD) rats specific pathogens free (SPF) were obtained from the Laboratory Animal Center of Ningxia Medical University. All handling and animal care procedures were conducted per Chinese National Institutes of Health guidelines and approved by the Medical Ethics Committee of Ningxia Medical University, China (2023-005). All animals were fed freely available food and sterile water at a controlled temperature of 22 ± 2 °C in a 12-h light/dark cycle environment. After one week in the experimental animal cage, the rats (180 ± 20 g) were randomly selected and divided into a control group (Control, n = 8) and a premature ovarian failure model group (POF, n = 40). The estrous cycle was detected using smears of vaginal shedding cells, and the estrous cycle of 96–120 h was selected for establishing subsequent animal models. In the POF group, rats were continuously injected with cyclophosphamide (CTX, Baxter, USA) intraperitoneally at a dose of 50 mg/kg/day on the first day, followed by intraperitoneal injection of CTX at a dose of 8 mg/kg/day for 14 consecutive days. In the control group, rats were injected with isodensity saline. After successful modeling, the POF group was randomly divided into the POF control group (POF, n = 20) and the stem cell treatment group (POF + MSCs, n = 20). The normal control group (Control) continued feeding. The well-grown fifth passage MSCs were collected and counted after washing with normal saline three times. The POF + MSCs group rats were transplanted with 1 × 10^6^/200 µL MSCs by tail vein injection at 14 days for the second transplantation. Rats in control and POF groups were injected with 200 µL of saline. After 28 days, rats were euthanized with CO_2_ suffocation, and the cardiac blood and ovarian tissue samples were collected for hormone tests and ovarian function measurements. The ovarian coefficient = (ovary weight/weight) and volume [mm^3^ = (π/6) × long (mm) × short (mm)^2^] were calculated [30]. Four rats from each group were randomly selected and mated with normal male rats for litter size counting.

### Ovarian culture in vitro

According to animal ethical requirements, three ovaries in each group (n = 3) were used in experiment of ovarian culture. Briefly, the bilateral ovaries were harvested from euthanized three-day-old suckling rats and washed with Phosphate Buffer Saline (PBS) for 10 s and then transferred to a sterile 35 mm plastic Petri dishes and completely submerged in DMEM/F12 high-glucose medium (Gibco) supplemented with 10% FBS, 5% insulin-transferrin-selenium, 1 mg/mL bovine serum albumin, 1 mg/mL albumin II, 100 µmol/L ascorbic acid, 1% penicillin-streptomycin, and 0.05 IU/mL follicle-stimulating hormone [31, 32]. The excess fat and connective tissues were removed from the ovaries using micro forceps under a stereoscope and then incubated at 37 °C and 5% CO_2_ for 30 min. CTX (60 µmol/L, Baxter, USA). VEGFA (50 ng/mL, PeproTech, USA) was added to the ovaries dishes for 48 h (n = 3), and then the ovaries were collected for subsequent experiments.

### Western blotting

The ovarian tissues or cells were incubated with Radio Immunoprecipitation Assay (RIPA) lysis buffer (P0013B, Beyond Biotech, Shanghai, China) containing protease inhibitors on ice for 30 min. After homogenizing, the samples were centrifuged at 12,000 rpm at 4 °C for 15 min, and the supernatant was collected in a new centrifuge tube for the protein concentration assay. The total protein concentration of the samples was detected using a bicinchoninic acid (BCA) Protein Assay Kit (KGP903, KeyGEN Biotech., Jiangsu, China). After boiling for 5 min, 40 µg of protein sample was loaded into each lane of a 12.5% sodium dodecyl sulfate-polyacrylamide gel, and electrophoresis was performed. The proteins were blotted onto a polyvinylidene difluoride (PVDF) membrane. Subsequently, the PVDF membrane was pre-incubated in a blocking buffer containing 5% nonfat milk containing 0.05% Tween 20 and then incubated with the appropriate primary and secondary antibodies. Finally, the protein expression signals were visualized using an enhanced chemiluminescence (ECL) western blotting detection kit (KGP1128, KeyGEN Biotech., Jiangsu, China) and a ChemiDocMP imaging system (Bio–Rad, USA). The gray value was recognized using ImageJ software. Assays were independently performed in triplicate for each sample. Antibodies against p-PI3K (p80α, AF3241) and PI3K (AF6241) were purchased from Affinity Biosciences Co., Ltd., USA; VEGFA (MAI-16,629) were purchased from Invitrogen Co., Ltd., USA; AKT (60203-2-Ig), mTOR (66888-1-Ig), and ATG5 (10181-2-AP) were purchased from Proteintech Co., Ltd., USA; P62 (ab109012) were purchased from Abcam. VEGFB (SC-80,442) was purchased from Santa Cruz Co., Ltd., USA; p-AKT (Ser473, #4060), p-mTOR (Ser2448, #2971), Beclin-1 (#3738), LC3A/B (#12,741) was purchased from Cell Signaling Technology Co., Ltd., USA; LC3B (D263557) was purchased from Sangon Biotech Co., Ltd, China; PCNA (10205-2-AP) and FSHR (22665-1-AP) were purchased from Proteintech Co., Ltd., China; CYP19A1 (A12684) and AMH (A8538) were purchased from ABclonal Technology Co., Ltd., China; α-tubulin (AF0001), β-actin (AF0003) were purchased from Beyond Biotech Co. Ltd., China; and all primary antibodies diluted at 1:1000.

### Histology analysis and follicle counting

The ovaries were fixed with 4% paraformaldehyde for 24 h, dehydrated with an alcohol gradient, and embedded in a paraffin embedding machine. A series of 5 μm tissue sections were prepared using a paraffin microtome, and one out of every five was stained with hematoxylin and eosin (H&E). Ovary morphology was observed and photographed under a light microscope. Finally, the number of primordial, primary, secondary, mature, and atretic follicles was counted.

### Immunohistochemistry and immunofluorescence

The ovarian tissue sections were deparaffinized with xylene and rehydrated with an ethanol concentration gradient. Endogenous peroxidase was blocked with 3% hydrogen peroxide, nonspecific binding with 2% BSA, and the antigen was restored with sodium citrate solution after heating. Subsequently, the sections were incubated with primary antibody (1:100) overnight at 4°C. After washing with PBS, the sections were incubated with a secondary antibody conjugated with horseradish peroxidase. Then, the protein expression was visualized using diaminobenzidine and hematoxylin staining, and the nuclei were stained with hematoxylin. After washing, the slices were sealed with neutral resin and dried in a fume hood. The positive cells were observed and evaluated using a photographic system and a light optical microscope. Analogously, the immunofluorescence was incubated with fluorescent secondary antibodies, and nuclei were stained with 4’,6-diamidino2-phenylindole (DAPI). Finally, the sections were sealed with an antifade solution and visualized using the confocal system (Nikon).

### Protein arrays assay

Serum samples (n = 4 each from POF and POF + MSCs) were collected from POF rats after two days of transplantation and sent to RayBiotech (RAT-Cytokine-Array-Q67, Guangzhou, China) for a biotin-label-based antibody array. The intensity values of signals from the plate for each sample were evaluated using a laser scanner. According to RayBiotech, normalization was done by multiplying the intensity values on each plate by the ratio of the positive controls on that plate to the mean of the positive controls on one randomly selected plate to adjust for plate-to-plate variation. The proteins with significant differences were subsequently analyzed for functional enrichment.

### Hormone level measurement

The anti-Müllerian hormone (AMH), follicle-stimulating hormone (FSH), estradiol (E2), and luteinizing hormone (LH) levels in serum were evaluated using commercially available enzyme-linked immunosorbent assay (ELISA) kits (Elabscience, Wuhan, China) according to the manufacturer’s instructions. Briefly, the serum was diluted 10 times, added to the 96-well plates containing the corresponding antibody, and incubated for 2 h. Then, the absorbance was recorded using a microplate reader and measured the hormone concentration in the serum. Finally, the hormone concentration was calculated in serum depending on the standard curve.

### Isolation and culture of primary GCs

Three-week-old female SD rats were purchased from the Experimental Animal Center of Ningxia Medical University. The rats were injected intraperitoneally with 10 IU of pregnant mare serum gonadotropin in saline and anesthetized after 48 h. The ovaries were isolated under aseptic conditions. After washing twice with PBS, the GCs were released after puncturing the follicles with a needle using a stereoscopic microscope. Then, the cell suspension was filtered through a 200 mesh cell strainer sieve, centrifuged at 1,000 rpm for 5 min, and re-suspended in DMEM/F-12 medium supplemented with 10% FBS and 1% penicillin/streptomycin. Then, the primary GCs suspension was seeded into six-well plates and incubated at 37 °C and 5% CO_2_. After 24 h, non-adherent cells were gently washed with PBS and removed [19]. The GCs were treated with 500 µM CTX and co-cultured with spherical MSCs in a Transwell system for administration. Then, GCs were collected for subsequent experiments.

### Assessment of oxidative stress

The oxidative stress level of tissues and cells was assessed using malondialdehyde (MDA, A003-1), superoxide dismutase (SOD, A006-2-1), and glutathione peroxidase (GSH, A001-3) kits (Nanjing Jiancheng Co., Ltd). The supernatant of ovarian homogenate and cell culture medium was collected using a microplate reader under the manufacturer’s instructions to detect the absorbance. Finally, the concentration or unit of each sample was calculated, and the oxidative stress effects were determined in ovaries and cells.

### Cell culture and cell viability assay

The human ovarian granulosa cell line KGN was purchased from the Shanghai Cell Bank of the Chinese Academy of Sciences. KGN was cultured in DMEM/F12 medium supplemented with 10% FBS and 1% penicillin/streptomycin incubated at 37 °C and 5% CO_2_. When the cell fusion rate reached 80%, cells were digested with 0.25% trypsin and passaged in new dishes. The cell viability was detected using a Cell Counting Kit-8 (CCK-8, APExBIO Technology, USA) according to the manufacturer’s instructions. Briefly, KGN cells or GCs were seeded in 96-well plates at a density of 8,000/well for 24 h, followed by incubation with 10% detection reagent for 2 h after CTX or MSCs co-culturing for 48 h. Each sample’s optical density value was determined using a microplate reader at 450 nm. The mean of three independent tests was calculated to determine the viability of KGN and GCs.

### 3D MSCs spheroid preparation

The hUC-MSCs at 90% density were digested with 0.25% trypsin and centrifuged at 1,000 rpm for 5 min. Then, the cells were re-suspended with medium, and concentrations were measured using a hemocytometer after adjusting the cell concentration to 2.5 × 10^4^/25 µL. Briefly, 25 µL cell suspension was dripped into a lid of a 10 cm petri dish and then gently placed on the petri dish with PBS. After culturing for 48 h, 3D MSCs spheroids were collected for co-cultured experiments [31].

### Autophagy flux detection

Primary GCs of 2 × 10^4^ were seeded on coverslips in a 24-well plate and incubated for 12 h at 37 °C and 5% CO_2_ before adding the mixture of mCherry-GFP-LC3B adenovirus (Beyotime, Shanghai, China) and pro-viral infection reagent Polybrene (Beyotime) for 24 h. After replacing with fresh medium, cells were treated with CTX, VEGFA, 3-MA, or/and rapamycin. The treated cells were fixed with 4% paraformaldehyde for 10 min and stained with DAPI for 10 min. After washing, the cells were sealed with an antifade solution and visualized using the confocal system (Nikon).

### Real-time quantitative polymerase chain reaction (RT-qPCR)

Total RNA was extracted from the ovaries using the RNAiso Plus kit (Invitrogen, USA). The concentration and quality of total RNA were measured using a NanoDrop 2000 (Thermo Scientific, USA). Reverse transcription and RT-qPCR quantification of RNAs were performed using a Quantscript RT Kit (TaKaRa) and 2× SYBR Green qPCR Master Mix (TaKaRa), respectively. *Lhx8*, *Nanos3*, *Lin28a*, *Nobox*, and *Bmp15* expression levels were analyzed using a RT-qPCR instrument (Bio-rad, USA). Gene-specific primers are presented in Table S1. The gene expression levels from three independent experiments were calculated using the 2^−ΔΔCt^ method.

### Statistical analysis

All data were analyzed using GraphPad Prism 6.0 software (San Diego, CA, USA). The data were visualized based on the means ± SD. Student’s t-tests and analysis of Variance (ANOVA) were used to determine the significant differences, and a *P*-value less than 0.05 was considered significant. Each experiment was repeated thrice.

## Results

### hUC-MSCs restored ovarian function and fertility in cyclophosphamide-induced POF rats

**Fig. 1:**
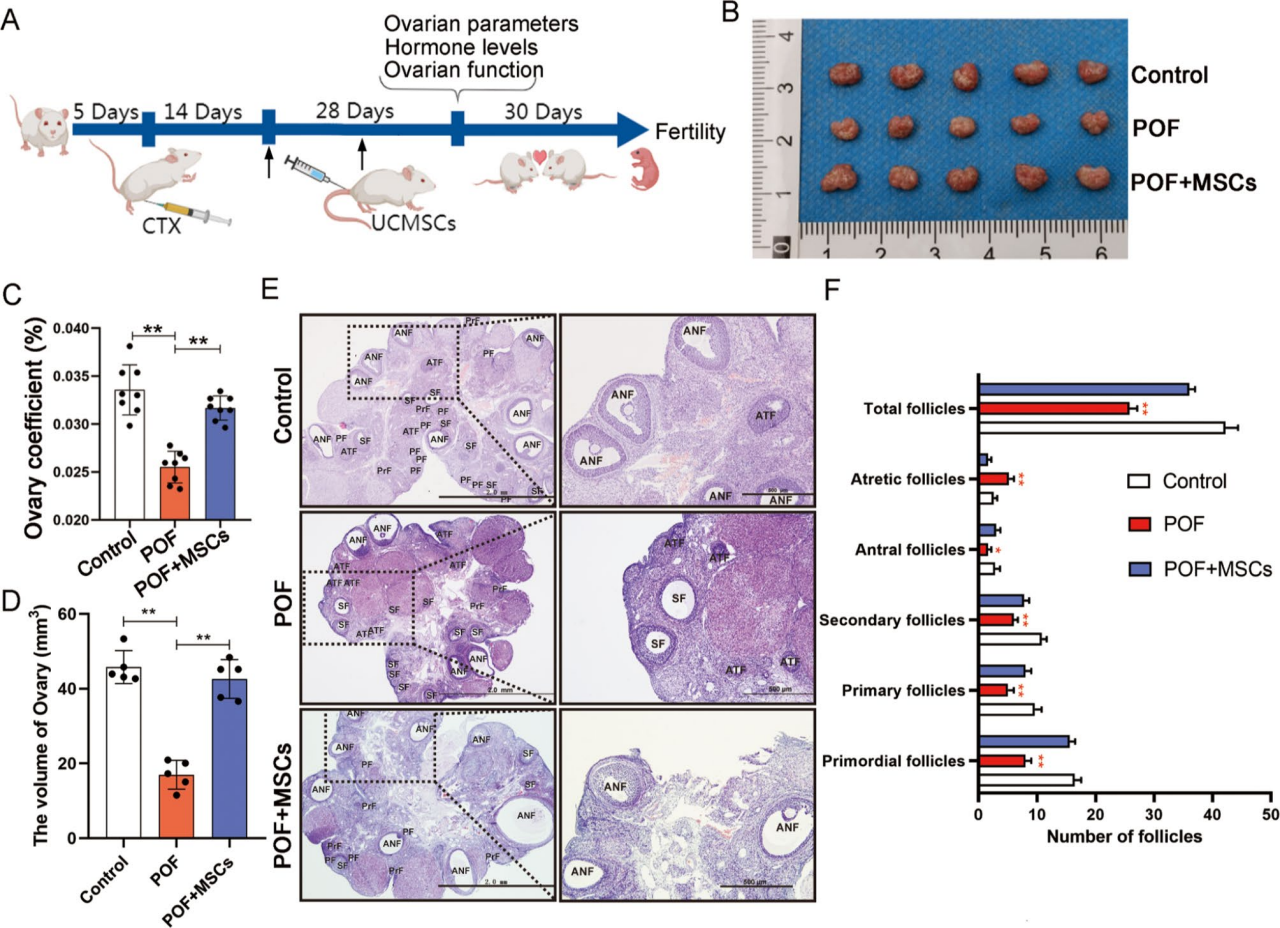
Recovery of ovarian morphology and structure in POF rats model using hUC-MSCs transplantation. **A** Schematic description of the experimental design. **B** Ovarian morphology. **C** Ovarian organ coefficient. **D** Ovarian volume. **E** Ovarian histology using H&E staining; scale bar: 2.0 mm and 500 μm. **F** Number of follicles (n = 5). *p < 0.05, **p < 0.01; ANF, antral follicles; ATF, antral follicle; CL, corpus luteum; SF, secondary follicle; PF, primary follicle; PrF, primordial follicle

**Fig. 2:**
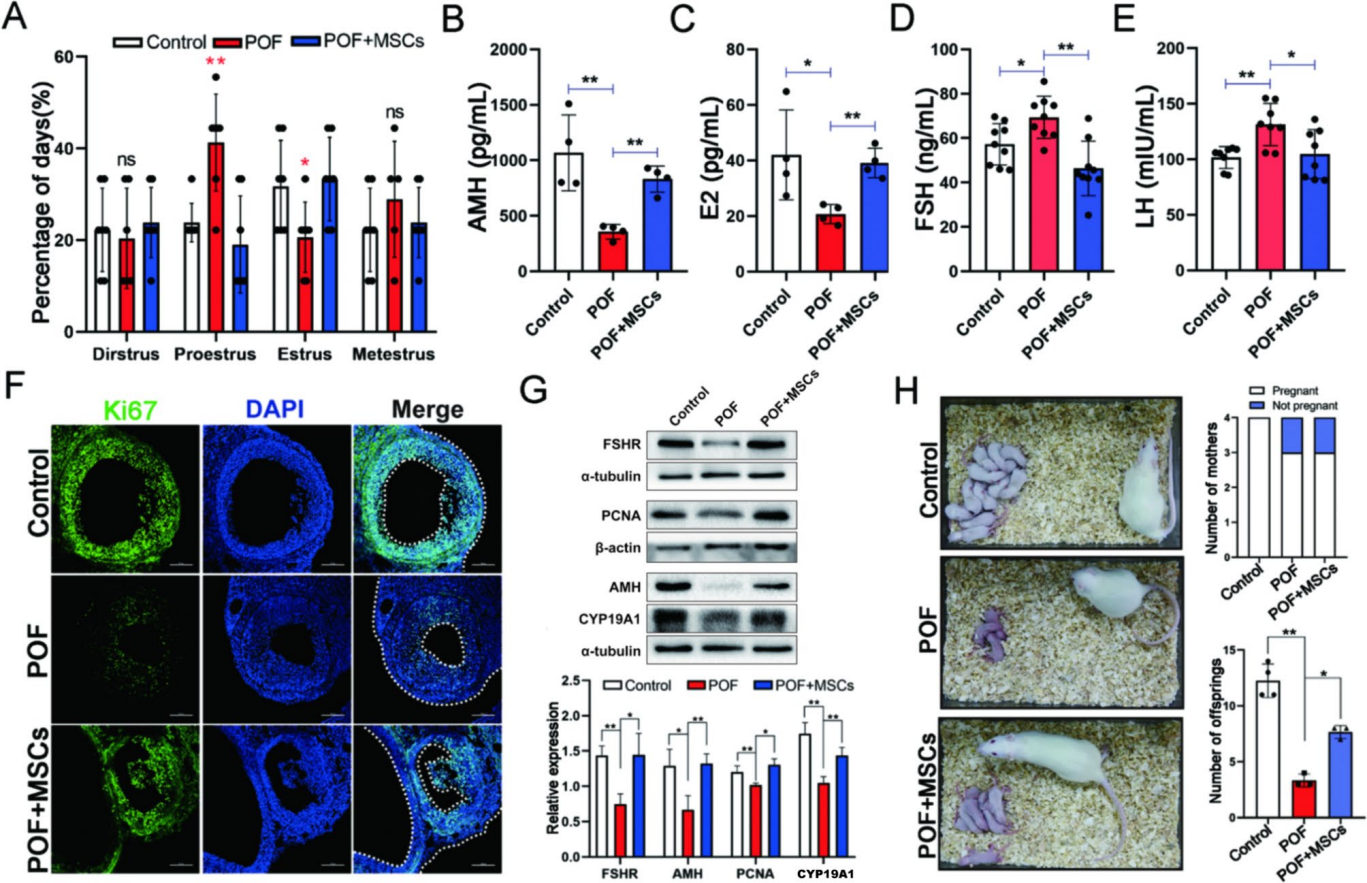
hUC-MSC improved the ovarian function of POF rats. **A** The percentage of each stage in the estrus cycle (the asterisk indicates there are significances in that group by comparing to the other two groups). The serum AMH (**B**), E2 (**C**), FSH (**D**), and LH (**E**) levels. **F** Ki67 expression in ovarian GCs; scale bar: 100 μm. **G** The Western blotting images and quantitation of functional protein expression in GCs. **H** The fertility and litter size. *p < 0.05. **p < 0.01

In our previous study, hUC-MSCs was isolated and identified [29]. The ovarian function and fertility were tested after hUC-MSCs injections twice to evaluate the effects of hUC-MSCs treatment on POF rats. Figure 1 A depicts the design flow of animal treatments (For convenience, hUC-MSCs were abbreviated to MSCs in all subsequent descriptions). Cyclophosphamide was administered intraperitoneally at a dose of 50 mg/kg/ day, followed by continuous administration at 8 mg/kg/ day for 14 days with normal feeding. After 15 days, experimental animals were randomly selected to test the establishment of the model, and the ovary was examined by paraffin section and HE staining for pathological observation (Fig. S2A), and the content of FSH and E2 in serum of rats was detected by ELISA (Fig. S2B and C). The criteria of POF model group were: FSH increased significantly, E2 decreased significantly; Compared with the control group, the number of follicles in the pathological section of ovary was reduced, the number of follicles was smaller and the number of granular cell layers was reduced. Regarding ovarian morphology, the POF group had significantly smaller ovaries than the control group, and the POF group recovered significantly more than the POF group after hUC-MSCs transplantation (Fig. 1B). Meanwhile, hUC-MSCs also improved ovarian organ coefficient and ovarian volume (Fig. 1C and D). Pathological assessments revealed that the numbers of total follicles, antral follicles, secondary follicles, primary follicles, and primordial follicles increased, while the number of atretic follicles was decreased in the POF + MSCs group than in the POF group (Fig. 1E and F). Subsequently, we evaluated the estrous cycle and hormone level. The results indicated that the proestrus and estrus phases were significantly improved after hUC-MSCs transplantation (Fig. 2A, Fig. S1A and S1B), while the AMH (Fig. 2B), E2 (Fig. 2C), FSH (Fig. 2D), and LH (Fig. 2E) levels were significantly changed compared to POF group. Ki67, a marker of cell proliferation, was detected by immunofluorescence staining to determine the regulated target of MSCs in ovarian function improvement. The result revealed that Ki67 expression was significantly higher in ovarian GCs of the POF + MSCs group than in the POF group (Fig. 2F, Fig. S2D). The functionally related proteins (FSHR, AMH, and Cyp19a1) of GCs and proliferation marker (PCNA) in the ovary were detected using western blotting to confirm further that GCs are the regulated targets of MSCs. The results indicated that the POF + MSCs group had significantly increased protein expression levels than the POF group. Additionally, fertility, including the number of pregnant mothers and offspring, was counted. The results exhibited that MSCs significantly improved the fertility of POF rats (Fig. 2H), suggesting that hUC-MSCs restored the ovarian function and fertility of POF by regulating GCs.

### hUC-MSCs reduced the excessive autophagy and oxidative damage of GCs in the POF rats model

**Fig. 3:**
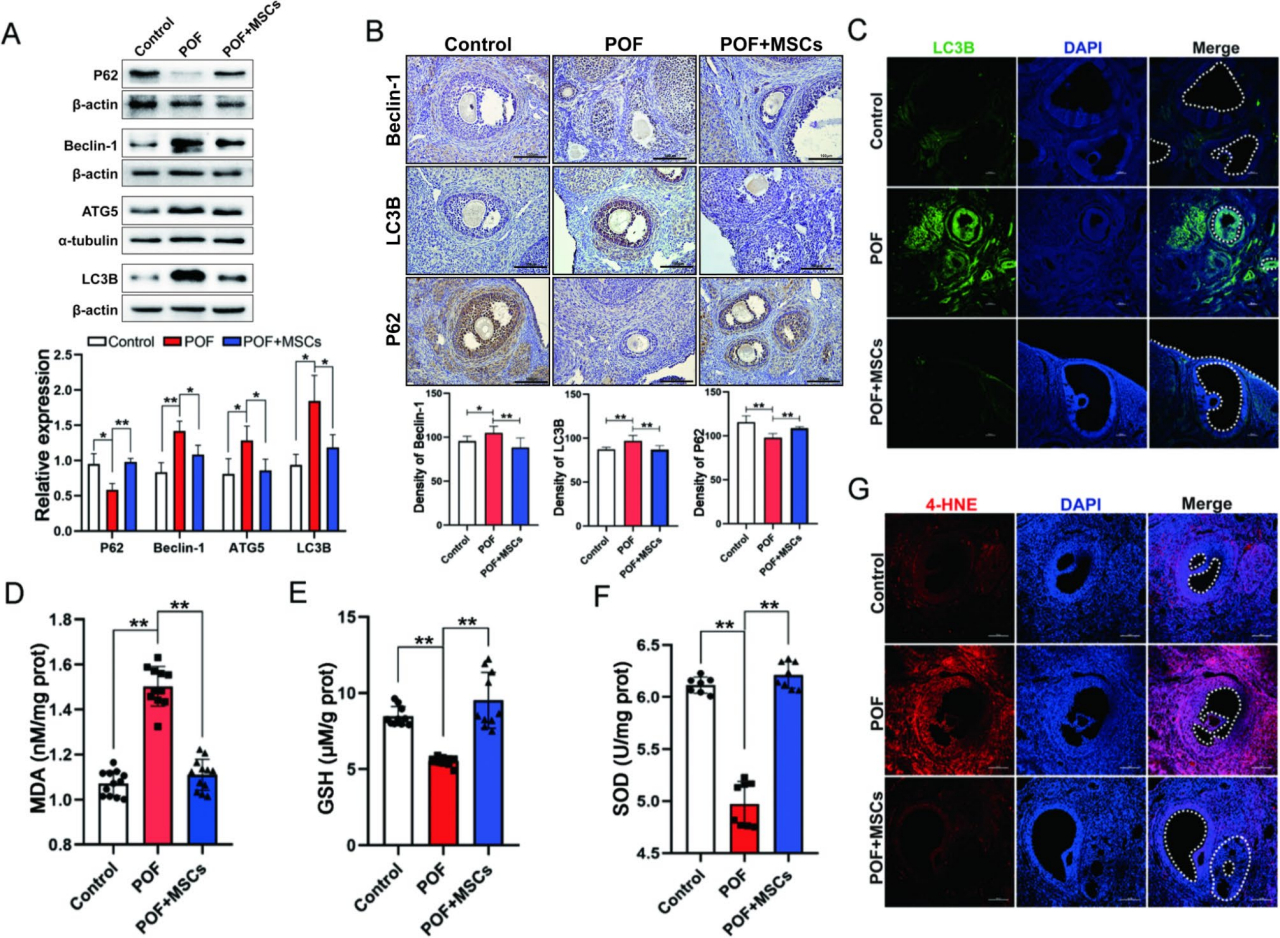
The oxidative damage and excessive autophagy in ovarian GCs were alleviated via hC-MSCs. **A** The Western blotting images and quantitation of autophagy associated proteins expression in ovaries. **B** The immunohistochemistry images and quantitation of autophagy-associated proteins expression in ovaries; scale bar: 100 μm. **C** The immunofluorescence images of LC3B expression in ovaries; scale bar: 100 μm. MDA (**D**), GSH (**E**), and SOD activity (**F**) levels in ovaries. **G** The lipid peroxidation producer 4-HNE in ovaries; scale bar: 100 μm. *p < 0.05. **p < 0.01

Numerous studies have reported that oxidative stress-induced autophagy of GCs is an important cause of follicular atresia and POF formation [19, 32]. The key autophagy protein expressions, including P62, Beclin-1, ATG5, and LC3B in ovaries, were detected using Western blotting. The results revealed that autophagy significantly changed the level in the ovary after MSCs transplantation (Fig. 3A). Similarly, compared to the POF group, the immune-histochemical results showed that P62, Beclin-1, and LC3B expressions in ovaries were also affected by MSCs, with changes in autophagy primarily occurring at GCs (Fig. 3B). Additionally, the immunofluorescence result exhibited that LC3B expression was decreased in POF + MSCs than in the POF group (Fig. 3C, Fig. S2E). Therefore, these results suggested that hUC-MSCs improved ovarian function by reducing the autophagy of GCs. The oxidative damage levels of ovarian tissue were detected to understand the causes of autophagy in GCs further. The results revealed that MDA, GSH, and SOD activity levels were significantly changed in the POF + MSCs group than in the POF group (Fig. 3D–F). Simultaneously, the end-product of lipid peroxidation 4-hydroxynonenal (4-HNE) was higher in the GCs of the POF group than control, while it was decreased in POF + MSCs group (Fig. 3G, Fig. S2F). These results indicated that hUC-MSCs could reduce the excessive autophagy and oxidative damage of GCs.

### hUC-MSCs activated PI3K/AKT/mTOR pathway by mediating VEGF in the ovary

**Fig. 4:**
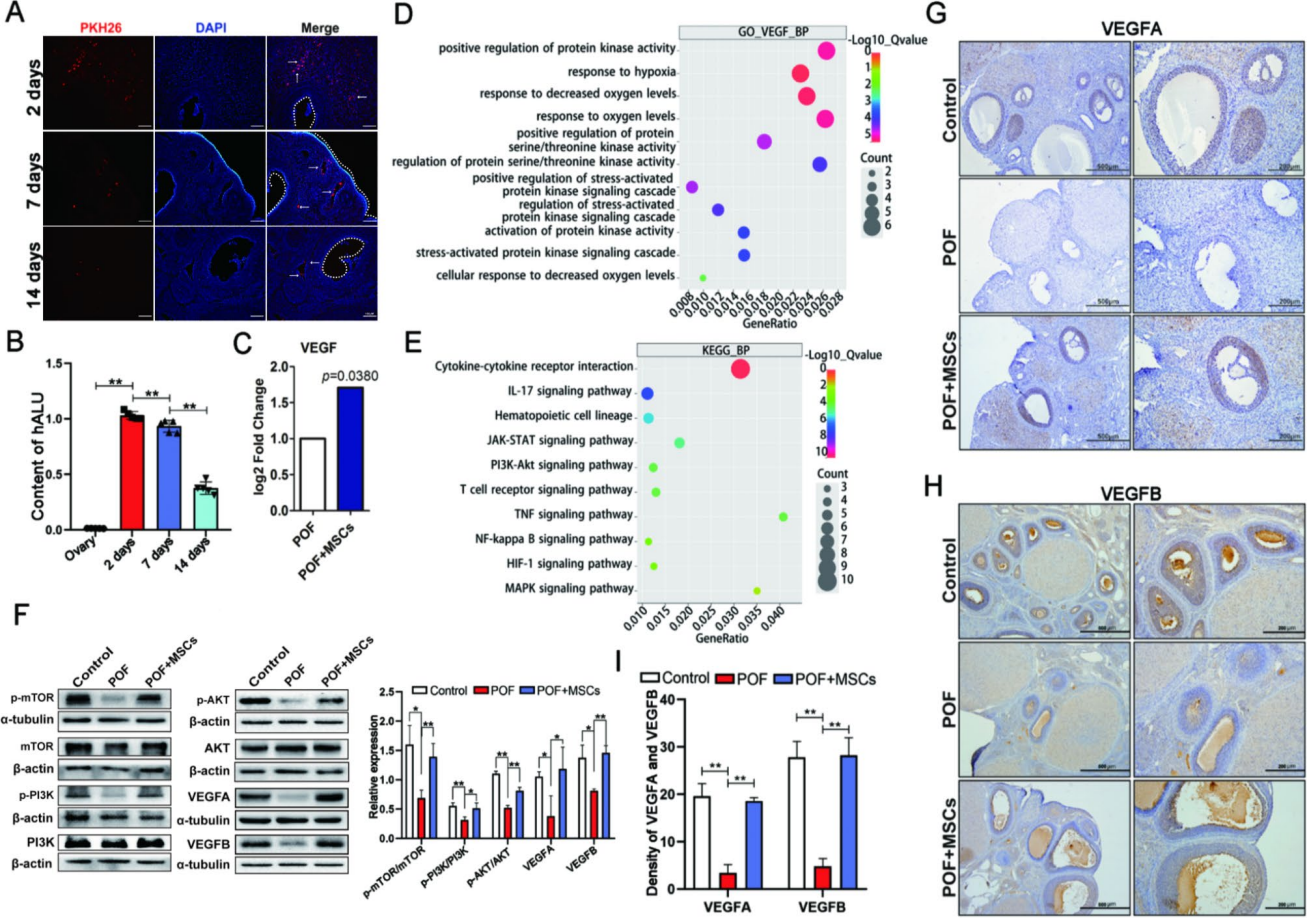
hUC-MSCs activated PI3K/AKT/mTOR pathway by mediating VEGF in the ovary; scale bar: 100 μm. **A** The localization of PKH26 labeled hUC-MSCs in the POF ovary after transplanting for 2, 7, and 14 days. **B** The human ALU gene levels in rat ovary after hUC-MSCs transplanting for 2, 7, and 14 days. **C** VEGF levels in serum after hUC-MSCs transplanting for two days. **D** The biological process of GO enrichment involved in VEGF regulating oxidative stress. **E** The signaling pathways of differential proteins enrichment after MSCs transplantation. **F** VEGF and PI3K/AKT/mTOR-related protein expressions in ovaries after MSCs transplantation. VEGFA (**G**) and VEGFB (**H**) expressions were determined in ovaries using immunohistochemistry; scale bar: 500 μm and 200 μm. **I** The quantification of VEGFA and VEGFB expression in **G** and **H**, respectively. *p < 0.05. **p < 0.01

PKH26-labeled hUC-MSCs were transplanted into the POF rat model via tail vein injection to determine the engraftment of hUC-MSCs in the ovary. The labeled MSCs were detected in ovaries at 2, 7, and 14 days after transplantation using a frozen section. The results demonstrated that the labeled MSCs gradually declined in ovaries over time (Fig. 4A), presenting a similar result by detecting the hALU levels (specific genes in human species) in rat ovaries (Fig. 4B). The rat’s serum two days after MSCs transplantation was used for protein microarray analysis to explore the mechanisms of MSCs in vivo in improving POF. The result exhibited that the VEGF level was significantly higher in the POF + MSCs group than in the POF group (Fig. 4C). The biological process of GO enrichment analysis involved the changes in VEGF regulation oxidative stress-related pathways (Fig. 4D). KEGG enrichment analysis results demonstrated that the PI3K/AKT signaling pathway was activated after MSCs transplantation. Therefore, VEGF (VEGFA and VEGFB) and PI3K/AKT/mTOR pathway-related protein expressions were detected using Western blotting in the ovary. The result revealed that the VEGFA, VEGFB, and PI3K/AKT/mTOR pathway expressions were significantly recovered in the POF + MSCs group compared to the POF and control groups (Fig. 4F). Furthermore, immunohistochemistry results exhibited that MSCs recovered VEGFA (Fig. 4G and I) and VEGFB (Fig. 4H and I) expressions in ovaries. GCs primarily reflected VEGFA and VEGFB expression differences between the three groups (Fig. 4G and H). These results suggested that hUC-MSCs regulated ovarian GCs via VEGF and PI3K/AKT/mTOR pathways.

Based on the above results, we supposed that hUC-MSCs might regulate excessive autophagy of GCs and improve ovarian function in the POF rat model via VEGF/PI3K/AKT/mTOR pathway.

### hUC-MSCs ameliorated excessive autophagy of GCs via paracrine in vitro

**Fig. 5:**
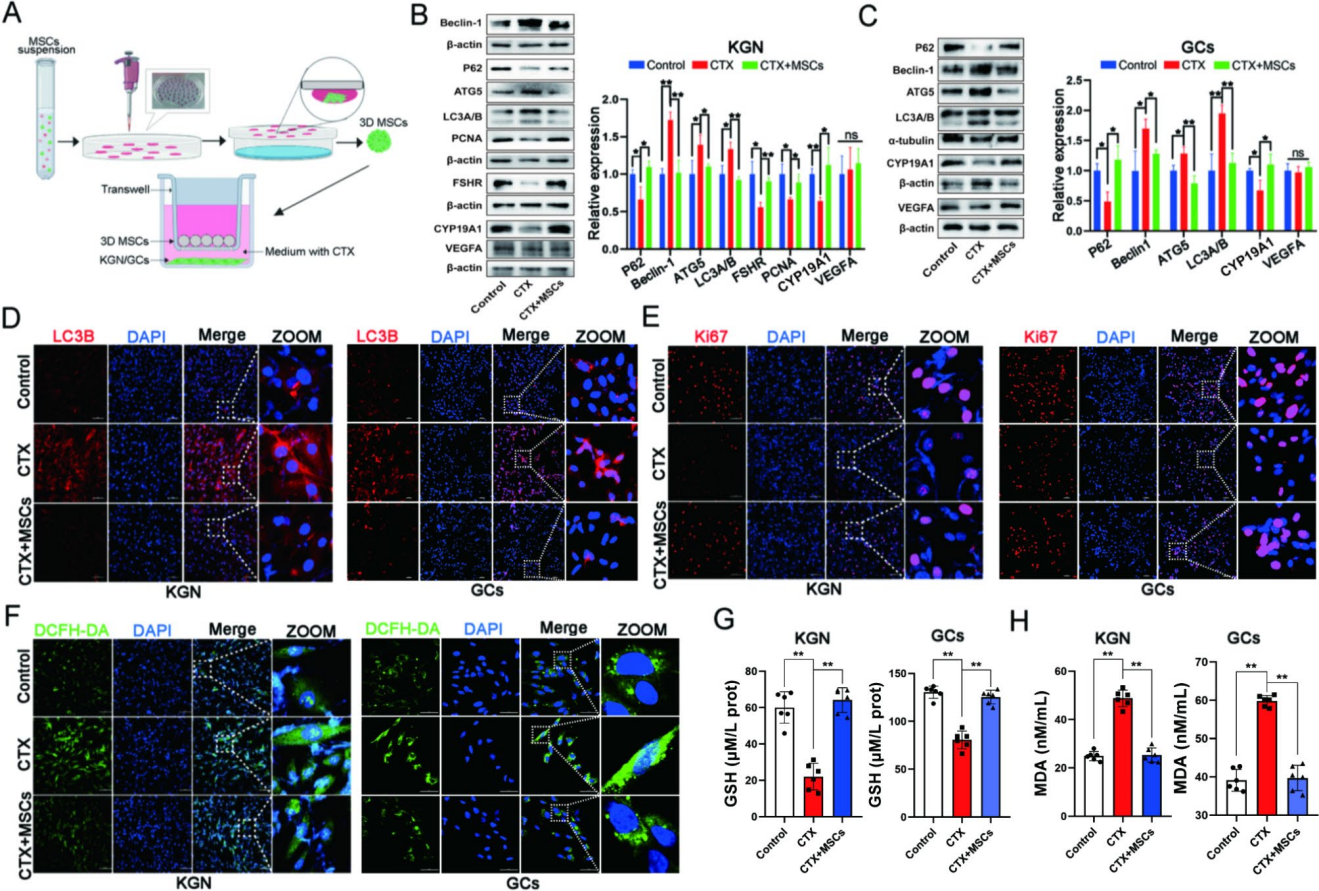
hUC-MSCs ameliorated excessive autophagy of GCs via paracrine in vitro. **A** Schematic diagram of 3D MSCs spheroids fabrication with KGN/GCs co-culturing. The autophagy-related proteins (P62, Beclin-1, ATG5, LC3A/B) and ovarian GCs function proteins (FSHR and Cyp19a1) expressions in KGN (**B**) and primary GCs (**C**), co-cultured with 3D MSCs spheroids. The autophagy protein LC3B (**D**) and proliferation protein Ki67 (**E**) expressions in KGN and primary GCs after co-culturing with 3D MSCs spheroids using immunofluorescence detection; scale bar: 100 μm in KGN and 50 μm in GCs. ROS (**F**), GSH (**G**), and MDA (**H**) levels in KGN and primary GCs after co-culturing with 3D MSCs spheroids. *p < 0.05. **p < 0.01

Previous studies have proved that 3D MSCs spheroids have a stronger paracrine ability than monolayer MSCs to simulate better the agglomerate state of MSCs in vivo and the ability to secrete cytokines that act indirectly on ovarian GCs [33]. Figure 5 A depicts a schematic of the indirect co-cultivation of MSC spheroids with CTX-induced KGN cell line or primary ovarian GCs via transwell plate. After treatment, the KGN/GCs in the bottom chamber were collected for determination. The Western blotting results indicated that hUC-MSCs could recover the changes of CTX-induced autophagy-related proteins (P62, Beclin-1, ATG5, and LC3A/B), function (FSHR and Cyp19a1) and proliferative (PCNA) proteins in KGN cells, but did not affect endogenous VEGFA expression (Fig. 5B). Similarly, the results were further demonstrated in the primary ovarian GCs of rats (Fig. 5C). Subsequently, the immunofluorescence results showed that hUC-MSCs could change the CTX-induced expression of autophagy and proliferation markers LC3B (Fig. 5D) and Ki67 (Fig. 5E) in KGN and GCs via the paracrine effect, respectively. Additionally, the oxidative damage s, including ROS (Fig. 5F), GSH (Fig. 5G), and MDA levels (Fig. 5H), were tested. The results revealed that the co-culturing of MSC spheroids alleviated the oxidative damage levels of KGN and GCs. Therefore, these results suggested that hUC-MSCs could restore CTX-induced excessive autophagy of GCs via the paracrine effect in vitro.

### Exogenous VEGFA alleviated excessive autophagy of GCs in vitro

**Fig. 6:**
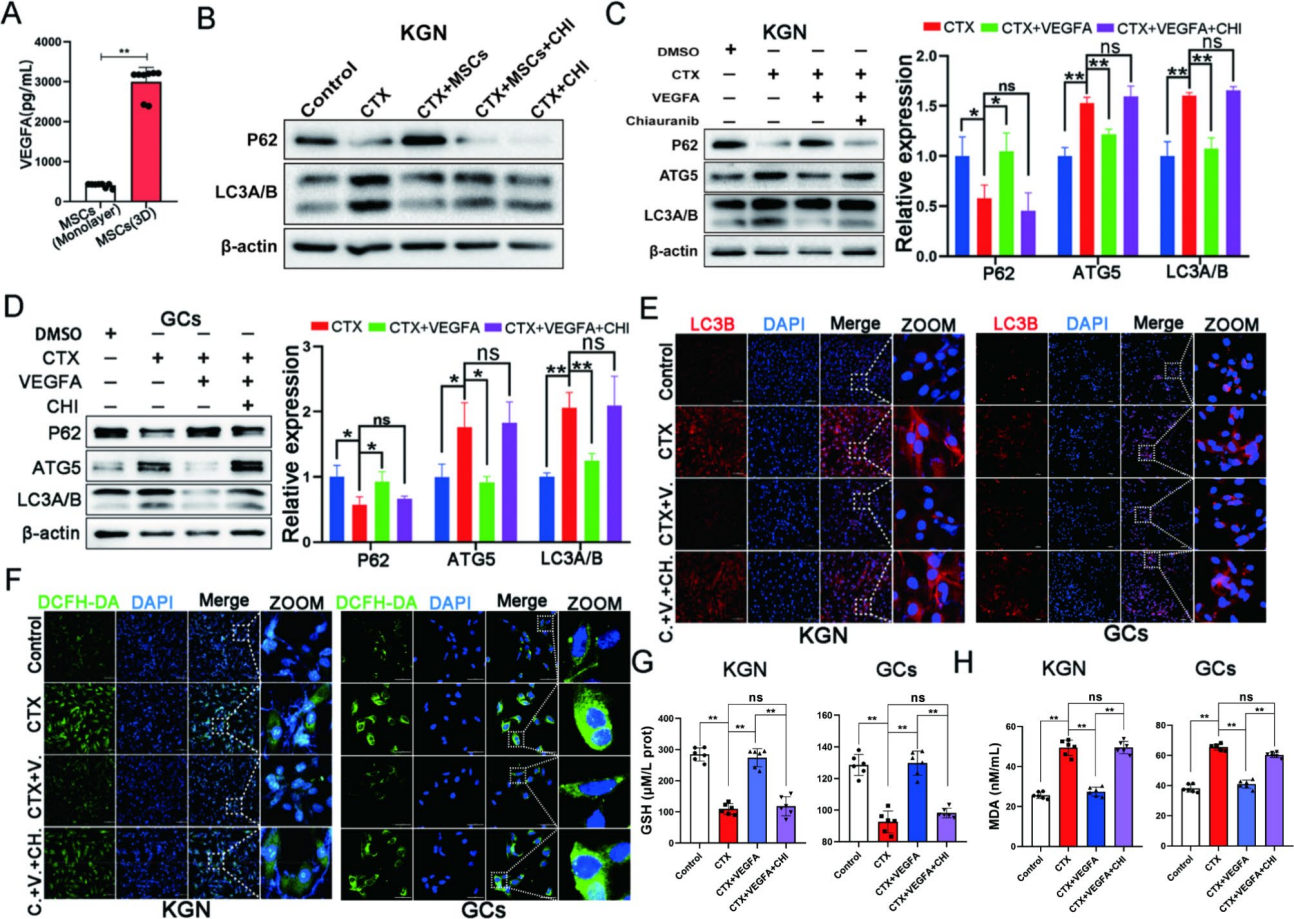
Exogenous VEGFA alleviated excessive autophagy of GCs in vitro. **A** The capacity of secretion VEGFA was compared between monolayer and MSCs spheroids at the same amount. **B** The autophagy protein expression was changed in the co-culture of MSCs and KGN by chiauranib, an inhibitor of the VEGFA receptor. The effects of chiauranib on VEGFA rescuing CTX-induced excessive autophagy proteins expression in KGN (**C**) and primary GCs (**D**). The effects of chiauranib on VEGFA rescuing CTX-changed LC3B expression (**E**) and ROS level (**F**) in KGN and primary GCs; scale bar: 100 μm in KGN and 50 μm in GCs. The effects of chiauranib on VEGFA rescuing CTX-changed the GSH (**G**) and MDA (H) levels in KGN and primary GCs. *p < 0.05. **p < 0.01

Based on the above results in vivo and in vitro, we speculated that hUC-MSCs might regulate the excessive autophagy of ovarian GCs and improve ovarian function by secreting VEGF. VEGFA and VEGFB expressions in monolayer and MSC spheroids were detected to clarify the type of VEGF secreted by hUC-MSCs. The result exhibited VEGFA level in MSC spheroids was highest among these groups. Thus, we considered that the VEGFA secreted by MSCs spheroids might play an important role in regulating the autophagy of GCs. We discovered that the VEGFA level secreted by MSCs spheroids was higher than monolayer by examining VEGFA and VEGFB levels secreted by an equal number of monolayer and MSCs spheroids (Fig. 6A, Fig. S1C-E). These results suggested that VEGFA, paracrine by MSCs spheroids, plays a key role in regulating the excessive autophagy of ovarian GCs. Therefore, chiauranib, an inhibitor of the VEGFA receptor, was used in the co-culture of MSC spheroids and KGN cells. The results revealed indirect co-cultured sphere MSCs could not reverse CTX-induced autophagy after chiauranib treatment (Fig. 6B, Fig. S1F). Exogenous cytokine VEGFA addition to KGN cells (Fig. 6C) and primary ovarian GCs confirmed this result (Fig. 6D). Immunofluorescence also revealed that VEGFA could decrease LC3B expression (Fig. 6E). Additionally, GSH and MDA levels in KGN and GCs reflected this phenomenon (Fig. 6F). These results further suggested that VEGFA plays an important role in ameliorating the excessive autophagy of GCs induced by CTX.

### VEGFA ameliorated excessive autophagy of GCs via PI3K/AKT/mTOR pathway

**Fig. 7:**
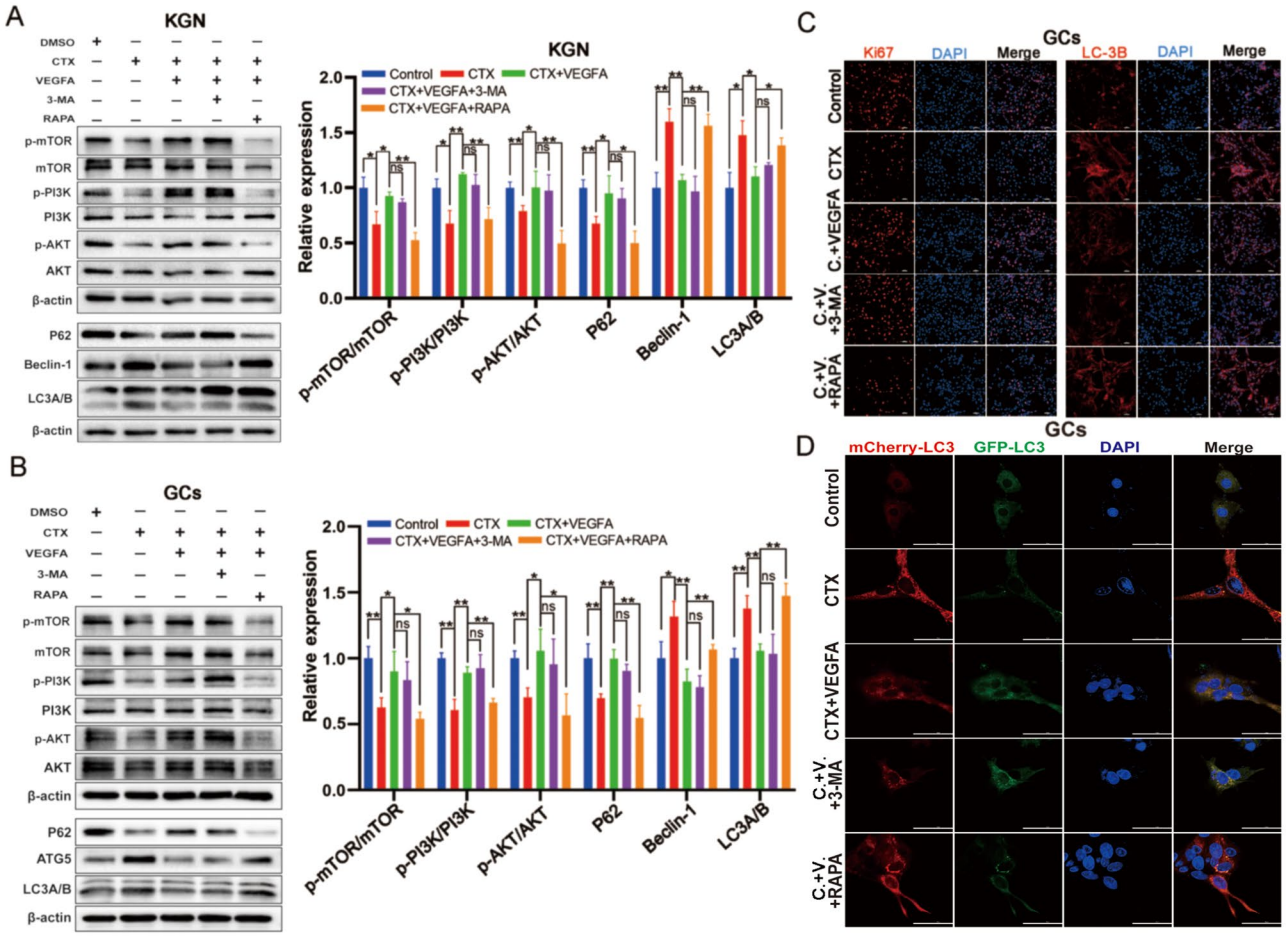
VEGFA ameliorated excessive autophagy of GCs via PI3K/AKT/mTOR pathway. The changes of autophagy and PI3K/AKT/mTOR signaling pathway related proteins expression were affected by VEGFA, 3-MA (autophagy inhibitor), and rapamycin (mTOR inhibitor) in KGN (**A**) and primary GCs (**B**). **C** The changes of Ki67 and LC3B expression were affected by VEGFA, 3-MA, and rapamycin in GCs; scale bar: 100 μm. **D** The changes in the autophagy flux were affected by VEGFA, 3-MA, and rapamycin in GCs; scale bar: 50 μm. *p < 0.05. **p < 0.01

Based on the above results in vivo and in vitro, we believed that VEGFA secreted by hUC-MSCs might regulate excessive autophagy of GCs via the PI3K/AKT/mTOR pathway. The inhibitor of autophagy (3-Methyladenine, 3-MA) and mTOR Rapamycin (RAPA) were employed to intervene in the process of VEGFA regulating excessive autophagy of KGN and GCs to demonstrate this hypothesis. The results indicated that VEGFA could ameliorate CTX-induced autophagy of GCs and activate PI3K/AKT/mTOR pathway. The 3-MA had no significant effect on the key proteins of the PI3K/AKT/mTOR pathway compared to the CTX + VEGFA group. However, the autophagy level (LC3II/I, P62, and Beclin-1) was increased, and PI3K/AKT/mTOR pathway was activated after RAPA addition in KGN (Fig. 7A) and primary GCs (Fig. 7B), compared to CTX + VEGFA group. Likewise, immunofluorescence of LC3B and Ki67 confirmed the autophagy and proliferation levels of primary GCs, respectively (Fig. 7C, Fig. S2I-J). Additionally, the autophagy flux was detected using plasmid transfected with mCherry-GFP-LC3B tandem fluorescent protein. The result demonstrated that CTX led to significant autophagy flux activation, which was blocked by VEGFA. The 3-MA did not change significantly autophagy flux activation, while RAPA supplementation activated autophagy flux (Fig. 7D). These results further demonstrated that VEGFA ameliorated excessive autophagy of CTX-induced GCs via PI3K/AKT/mTOR pathway.

### VEGFA improved excessive autophagy of GCs via PI3K/AKT/mTOR in ovarian culture in vitro

**Fig. 8:**
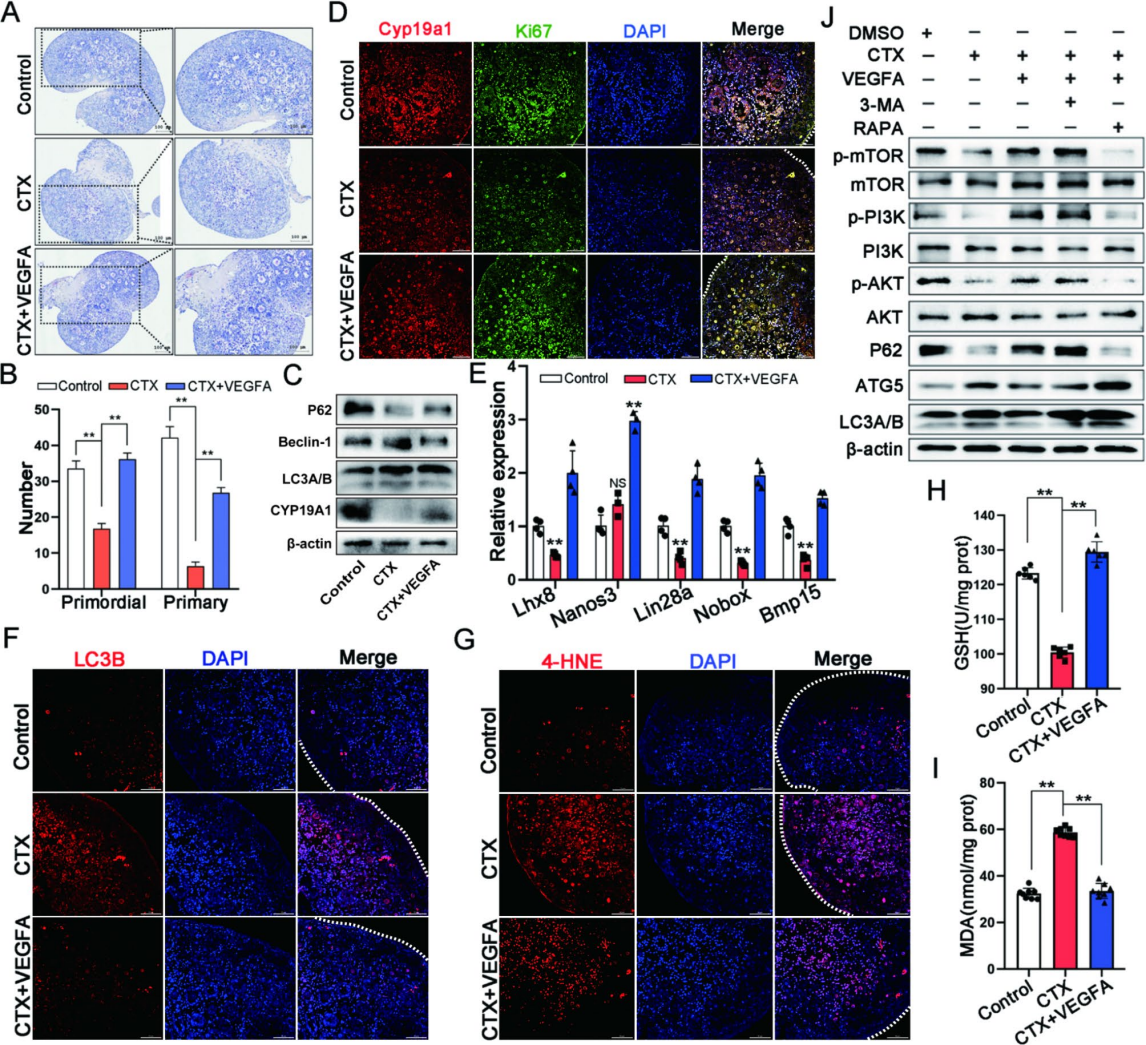
VEGFA improved excessive autophagy of GCs via PI3K/AKT/mTOR in ovarian culture in vitro. **A** The histologic features of the ovary in CTX damaging three-day-old rats rescued by VEGFA; scale bar: 100 μm. **B** The number of primordial and primary follicles in ovaries of Control, CTX, and CTX + VEGFA groups. **C** The autophagy proteins (P62, Beclin-1, and LC3A/B) and marker protein (Cyp19a1) expressions in GCs in ovaries among Control, CTX, and CTX + VEGFA groups. **D** Cyp19a1 and Ki67 expressions in ovaries among Control, CTX, and CTX + VEGFA groups by immunofluorescence detection; scale bar: 50 μm. **E** The effects of follicle development related genes *Lhx8*, *Nanos3*, *Lin28a*, *Nobox* and *Bmp15* in transcriptional level among Control, CTX and CTX + VEGFA groups. **F** The autophagy protein LC3B expression in ovaries among Control, CTX, and CTX + VEGFA groups via immunofluorescence detection; scale bar: 50 μm. **G** The levels of lipid peroxidation products 4-HNE in ovaries among Control, CTX, and CTX + VEGFA groups; scale bar: 50 μm. The oxidative stress markers GSH (**H**) and MDA (**I**) levels in ovaries among Control, CTX, and CTX + VEGFA groups. *p < 0.05. **p < 0.01

The ovaries of three-day-old lactating rats were treated with CTX and VEGFA to demonstrate further that VEGFA regulated excessive autophagy of ovarian GCs via PI3K/AKT/mTOR signaling. After 48 h of in vitro culture, the CTX + VEGF group had a significantly higher number of viable primordial and primary follicles than the CTX group (Fig. 8A and B). VEGFA could restore CTX-induced key autophagy protein (P62, Beclin-1, and LC3A/B) expressions and change Cyp19a1 expression, a functional marker of ovarian GCs, compared to CTX and control groups (Fig. 8C). VEGFA ameliorated Cyp19a1 and Ki67 expressions in CTX, reducing ovarian GCs function by immunofluorescence (Fig. 8D). *Lhx8*, *Nanos3*, *Lin28a*, *Nobox*, and *Bmp15* expressions, key genes of follicular development, were tested using qRT-PCR to confirm the effects on follicular development further. The results demonstrated that VEGFA improved the CTX-induced decrease of these genes. Meanwhile, immunofluorescence determined LC3B (Fig. 8F) and 4-HNE (Fig. 8G) levels. The results exhibited that VEGFA could restore CTX-induced changes in GSH (Fig. 8H) and MDA (Fig. 8I) levels. Additionally, the autophagy inhibitor 3-MA and mTOR inhibitor RAPA were used for treatment based on that VEGFA improves excessive autophagy of GCs in ovarian culture in vitro. The results revealed that 3-MA did not significantly change autophagy proteins (P62, ATG5, and LC3A/B), and PI3K/AKT/mTOR-related proteins expressions compared to VEGFA that improved CTX-induced excessive autophagy. In contrast to the VEGFA + CTX group, the autophagy level (LC3II/I, P62, and Beclin-1) was increased, and RAPA addition affected PI3K/AKT/mTOR pathway, consistent with the results in cell level (KGN and GCs). Therefore, these results further demonstrated that VEGFA improved ovarian function by ameliorating the excessive autophagy of GCs via PI3K/AKT/mTOR signaling pathway.

## Discussion

Numerous studies have reported that various MSCs are effective in treating POF. This study’s results indicated that the hUC-MSCs isolated from the umbilical cord were used to treat the POF rat model and exhibited a significantly improved effect, consistent with other reports regarding bone marrow and adipose-derived MSCs [12, 34]. Besides being noninvasive, safe, easily acquired, and without ethical debate, the hUC-MSCs obtaining procedure has several advantages, like relatively low immunogenicity and high paracrine efficacy due to neonatal tissue donors. Moreover, the process of hUC-MSCs improving POF involves the control of ovarian fibrosis, angiogenesis, immune system modulation, and apoptosis. Therefore, hUC-MSCs are a promising choice for improving the ovarian function POF due to these properties. In this study, the hUC-MSCs were applied to the POF rat model to study the mechanism further. The hUC-MSCs had been identified based on morphology, surface marker expression, and tri-lineage differentiation potential, described in our previous study [29]. Regarding the effect of the hUC-MSCs treated POF rat model, studies demonstrated that hUC-MSCs restored ovarian function by recovering disturbed hormone secretion, promoting folliculogenesis, and decreasing GCs apoptosis, consistent with our results [35–38]. Additionally, the hUC-MSCs transplantation approach used in these studies was intravenously administered, and the count of transplantation was 0.25–5 × 10^6^, similar to ours. In this study, two times 1 × 10^6^ hUC-MSCs each were injected into POF rats in a 14-day interval because the assay of PKH26 fluorescence-labeled hUC-MSCs in vivo discovered almost no labeled MSCs after 14 days. Furthermore, these PKH26 labeled MSCs primarily located in the ovarian interstitium suggested that hUC-MSCs did not differentiate into follicular cells or GCs. The mechanisms of hUC-MSCs restoring ovarian function may be related to its paracrine effect by improving the local microenvironment of the ovary [39]. After 28 days of the initial transplantation, FSH and LH levels decreased, while AMH and E2 levels increased, similar to a study of human placenta-derived MSCs treated POF [40]. FSH and LH are gonadotropic hormones secreted by the adenohypophysial gland that act on ovarian GCs to regulate estrogen secretion and follicle development. Nevertheless, with a limited increase in estrogen levels, serum FSH and LH levels may be decreased by negative feedback regulation of the hypothalamic-pituitary-gonadal axis to reduce follicle excessive growth and avoid their depletion [41]. Additionally, AMH, a factor of transforming growth factor β (TGF-β) superfamily, participates in initiation and dominant follicle recruitment and plays an important role in the follicular development of primordial follicle to the preantral follicle, and prevents rapid and premature depletion of follicles as well as preserves ovarian reserve function [42]. Therefore, this study’s results indicated that hUC-MSCs could successfully recover ovarian function and negative feedback regulation of hypothalamic-pituitary-gonadal in the POF model. In this study, hUC-MSCs was transplanted by tail vein. In our preliminary study, we found that intra-ovarian injection dependent invasive procedures greatly affect the evaluation of the therapeutic effect of MSCs (data not shown). Although 3D MSCs spheroids have potential therapeutic advantages, there is a risk of embolization via tail vein transplantation for small animals such as rodents, thus, intra-ovarian transplantation of MSCs spheroids may become the future development direction [43, 44]. In addition, MSCs derived exosomes have many advantages and may become a trend in this field [9].

This study determined that hUC-MSCs reduced ovarian oxidative stress levels and autophagy of GCs in vivo, and hUC-MSCs reduced the excessive autophagy of GCs via paracrine effects at the cellular level in vitro. Autophagy of ovarian GCs could be a double-edged sword during follicular development. The proper course of autophagy maintains cellular homeostasis and provides cytoprotection against stress-induced apoptosis. However, excessive autophagy (beyond a certain threshold) could destroy cytoplasm and organelles, leading to irreversible cell death [45]. Studies have exposed that excessive autophagy caused by oxidative stress may contribute to “autophagic death” and inhibiting excessive autophagy can attenuate the damage of ovarian GCs [17, 18]. For example, the normal autophagy of GCs plays an important role in developing dominant follicles. However, excessive autophagy of GCs will result in follicular atresia and ovarian dysfunction when stimulated by pollutants and substances that cause oxidative stress [46, 47]. Moreover, a study similar to our results showed that genetically modified hUC-MSCs restored the ovarian function of POF mice by reducing the autophagy of GCs [20]. Therefore, we believed that alleviating the excessive autophagy and oxidative stress levels of GCs might mainly contribute to the paracrine effects of MSCs because hUC-MSCs have a high cytokine secretion profile; it can secrete various cytokines with excellent antioxidant properties and promote cell survival [11]. In this study, autophagy-related proteins (ATG5, Beclin-1, LC3B, and P62) expressions were significantly reversed in the ovary of POF by MSCs transplantation. ATG5 and Beclin-1 are essential to the induction of autophagic occurrence; the formation of LC3B from LC3A indicates autophagy, P62 is the substrate of autophagy, and the expression is negatively correlated with autophagy [45]. This study also used mCherry-GFP-LC3, an autophagy flux monitoring system, at the cellular level. The mCherry-GFP-LC3 fusion protein is yellow fluorescence in the initial autophagy, and the GFP fluorescence was quenched by an acidic environment of autophagic lysosome formation, leaving only red fluorescence of mCherry [48]. We discovered that VEGFA might play a critical role in improving ovarian function via PI3K/AKT pathway using protein microarray assay to understand better the mechanisms by which hUC-MSCs improve POF, further verified in ovary tissue. Similar to our results, a study reported that hUC-MSCs restored the ovarian function of the POI model by activating PI3K/AKT signaling pathway and promoting VEGF expression [49]. Moreover, a study revealed that bone marrow-derived MSCs ameliorated renal damage by activating the PI3K/AKT signaling pathway and increasing serum VEGF levels [50]. Although the significant changes in VEGFA and VEGFB in the ovary of POF after MSCs transplantation, we chose VEGFA for later study in vitro because hUC-MSCs spheroids can secrete more VEGFA. This indicated that VEGFA is critical in improving vascular tissue remodeling by hUC-MSCs, similar to existing studies [51, 52]. The ovary’s vascular system must be remodeled to allow for the necessary nutrient, oxygen, and hormonal support during follicle and corpus luteum formation, as well as selecting the dominant follicle from the primary follicle [53, 54].

The phosphoinositide 3-kinase (PI3K)/protein kinase B (AKT)/mammalian target of the rapamycin (mTOR) signaling pathway plays an important role in inducing autophagy. Studies have revealed that the PI3K-AKT pathway inhibits autophagy via activating mTOR [55, 56]. Meanwhile, numerous studies have demonstrated that the PI3K-AKT pathway is critical for MSCs to improve ovarian function via a paracrine mechanism [57, 58]. However, the detailed mechanisms of this regulation remain uncertain. In the previous study, we discovered that metformin inhibited excessive autophagy of GCs via PI3K/AKT/mTOR pathway [17]. A study reported that PI3K/AKT/mTOR signaling pathway was required for FSH regulating oxidative stress-induced autophagy of GCs [19]. Therefore, based on these studies, we hypothesized that the paracrine VEGFA of hUC-MSCs alleviates excessive autophagy of GCs via PI3K/AKT/mTOR signaling pathway. Furthermore, it was confirmed by a series of experiments, including a non-contact co-culture system and the addition of inhibitors (chiauranib, 3-methyladenine, and rapamycin) in vitro. The results were consistent with existing studies [49, 59]. Although chiauranib is a multi-target inhibitor against aurora B kinase, VEGFR, colony-stimulating factor 1 receptor, platelet-derived growth factor receptor in tumor angiogenesis [60], the results of MSCs and VEGFA co-culture with KGN and GCs revealed that chiauranib targeted VEGFR2 on the cell membrane, and suggested that paracrine VEGFA of hUC-MSCs mitigated the CTX-induced excessive autophagy. According to a report, VEGF binds to the membrane surface receptor VEGFR2 and activates several intracellular signaling pathways, including PI3K/AKT pathway [61]. This study investigated the inhibitory effects of 3-MA and rapamycin on autophagy occurrence and mTOR regulation, respectively. The results indicated that VEGF regulates the excessive autophagy of KGN/GCs through PI3K/AKT/mTOR. However, the changes in key autophagy proteins did not completely consistent with the phosphorylation levels of PI3K/AKT/mTOR because the 3-MA used in this study inhibits the occurrence of autophagy via PI3K, implying that other signaling pathways are partially activated in autophagy suppression [62].

This study has several limitations. First, hUC-MSCs have a strong ability to secrete trophic factors, including VEGFA, while we only combined the findings in vivo and focused on the research of VEGFA regulating excessive autophagy of ovarian GCs. However, whether other factors or exosomes secreted by hUC-MSCs have the potential to regulate GCs, excessive autophagy remains to be further explored. Second, our study only confirmed that VEGFA secreted by hUC-MSCs alleviates excessive autophagy of GCs in vivo; whether other factors secreted by hUC-MSCs stimulate the ovary itself to secrete VEGFA, thus, improving ovarian function in vivo remains unknown. Third, as we know, tumor growth is positively correlated with angiogenesis. Our data exposed the VEGFA level was increased in blood circulation after hUC-MSCs transplantation; whether the high VEGFA levels can cause cancer development remains unclear. Thus, the safety of hUC-MSCs and VEGFA in clinical trials must be confirmed.

**Fig. 9:**
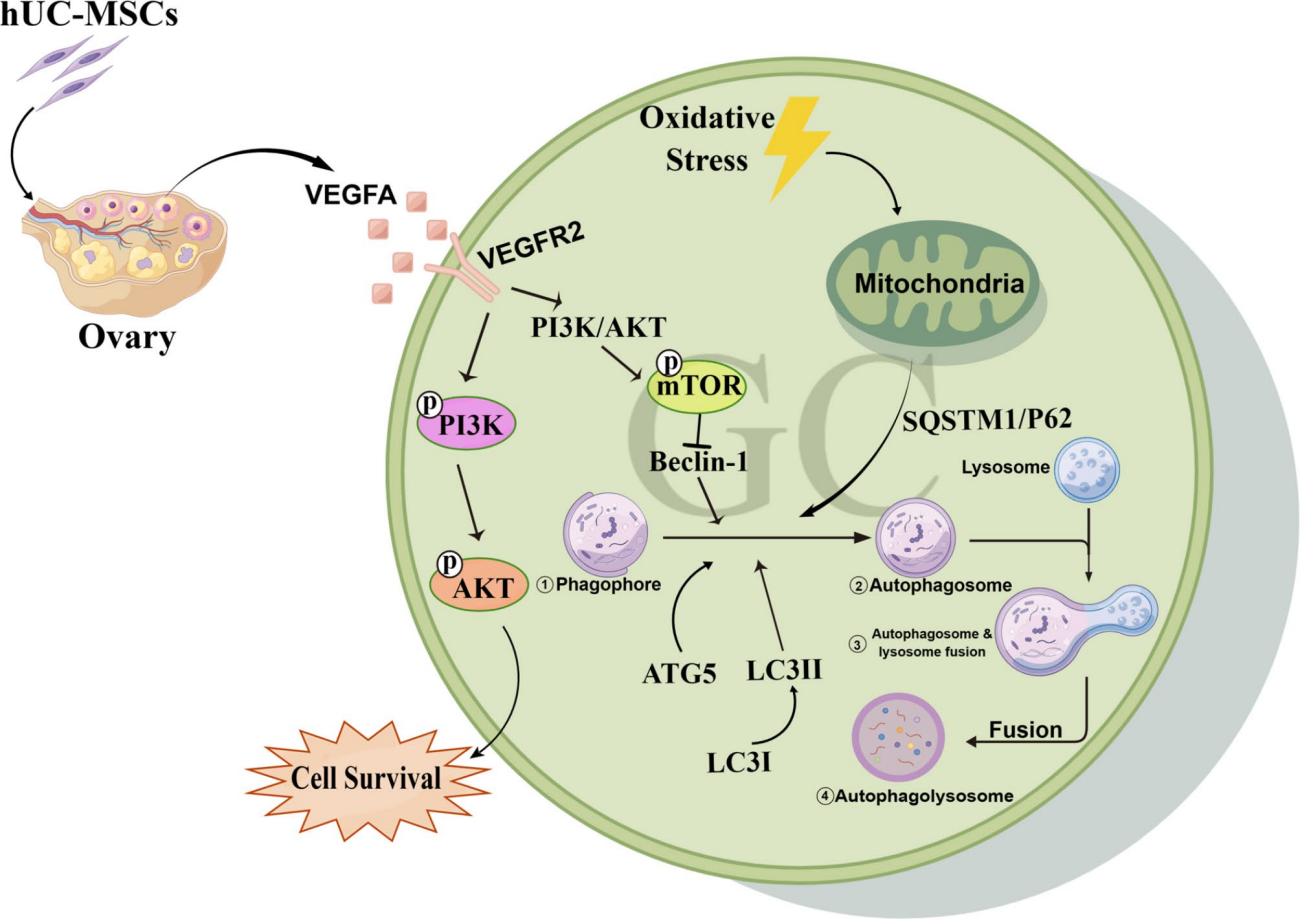
The schematic diagram of the potential mechanism in hUC-MSCs improving POF.

## Conclusion

The present study demonstrates that hUC-MSCs could alleviate excessive autophagy of ovarian GCs by secreting VEGFA and activating the PI3K/AKT/mTOR pathway, restoring ovarian function in cyclophosphamide-induced POF rats model (Fig. 9). Moreover, our study provides a novel insight into the potential mechanism of hUC-MSCs therapy for POF and a theoretical foundation for targeting angiogenesis therapy of POF and ovarian aging in the clinic.

### Electronic supplementary material

Below is the link to the electronic supplementary material.

Supplementary Material 1

## Data Availability

All data and materials are available in the manuscript, further inquiries can be directed to the corresponding author.

## Abbreviations

AKT: Protein kinase B
AMH: anti-Müllerian hormone
BCA: bicinchoninic acid
CCK-8: Cell counting iit-8
CTX: cyclophosphamide
ECL: enhanced chemiluminescence
E2: estradiol
FSH: follicle-stimulating hormone
GSH: glutathione peroxidase
H&E: hematoxylin and eosin
hUC-MSCs: human umbilical cord-derived mesenchymal stem cells
LH: Luteinizing hormone
MDA: malondialdehyde
mTOR: mammalian target of the rapamycin
PI3K: Phosphoinositide-3 kinase
POF: Premature ovarian failure
PVDF: polyvinylidene difluoride
RIPA: Radio immunoprecipitation assay
RT-qPCR: Real-time quantitative polymerase chain reaction
SOD: superoxide dismutase
VEGFA: vascular endothelial growth factor A
4-HNE: 4-hydroxynonenal
