# Supplementary material for: Human umbilical cord-derived mesenchymal stem cells (hUC-MSCs) alleviate excessive autophagy of ovarian granular cells through VEGFA/PI3K/AKT/mTOR pathway in premature ovarian failure rat model
Source: J Ovarian Res. 2023 Sep 30;16:198. doi: 10.1186/s13048-023-01278-z (PMC10542694; doi:10.1186/s13048-023-01278-z)
Supplement: Supplementary file 1 — Supplementary Material 1 [file 13048_2023_1278_MOESM1_ESM.docx]

| Target gene | Sequence (5’ 🡪 3’) | Size of amplicon (bp) | Species |
| --- | --- | --- | --- |
| F-Lhx8 | GTATCACTTGGCTTGCTT | 18 | Rat |
| R-Lhx8 | ATTACCGTTCTCCACTTC | 18 |  |
| F-Nanos3 | CTCTGCATGAGGAAGAGGAGCC | 22 | Rat |
| R-Nanos3 | GGACTGATAGATCGCACGAGA | 21 |  |
| F-Lin28a | CCCGGTGGACGTCTTTGTG | 19 | Rat |
| R-Lin28a | CACTGCCTCACCCTCCTTGA | 20 |  |
| F-Nobox | AGCCAGTGCAGATCTGCACC | 20 | Rat |
| R-Nobox | TGTCACTGCCAGGAACATCCCTC | 23 |  |
| F-BMP15 | ATCTGATGTCCCTTGTCCTT | 20 | Rat |
| R-BMP15 | CTCTGTATTGATGGCATGGTT | 21 |  |
| VEGFB-F | AGTGCTGTGAAGCCAGACA | 19 | Human |
| VEGFB-R | GGAGTGGGATGGGTGATG | 18 |  |
| VEGFA-F | TGCGGATCAAACCTCACCA | 19 | Human |
| VEGFA-R | CAGGGATTTTTCTTGTCTTGCT | 22 |  |
| ALU-F | AGACCATCCTGGCTAACACG | 20 | Human |
| ALU-R | AGACGGAGTCTCGCTCTGTC | 20 |  |
| GAPDH-F | CACCACACTGAATCTCCCCTC | 21 | Human |
| GAPDH-R | TCCCCAGCAAGAATGTCTCA | 20 |  |
| GAPDH-F | AAGGAGTAAGAAACCCTGGACC | 22 | Rat |
| GAPDH-R | CTCCTGTTGTTATGGGGTCTGG | 22 |  |

**Table S1 Primers for real-time qPCR**


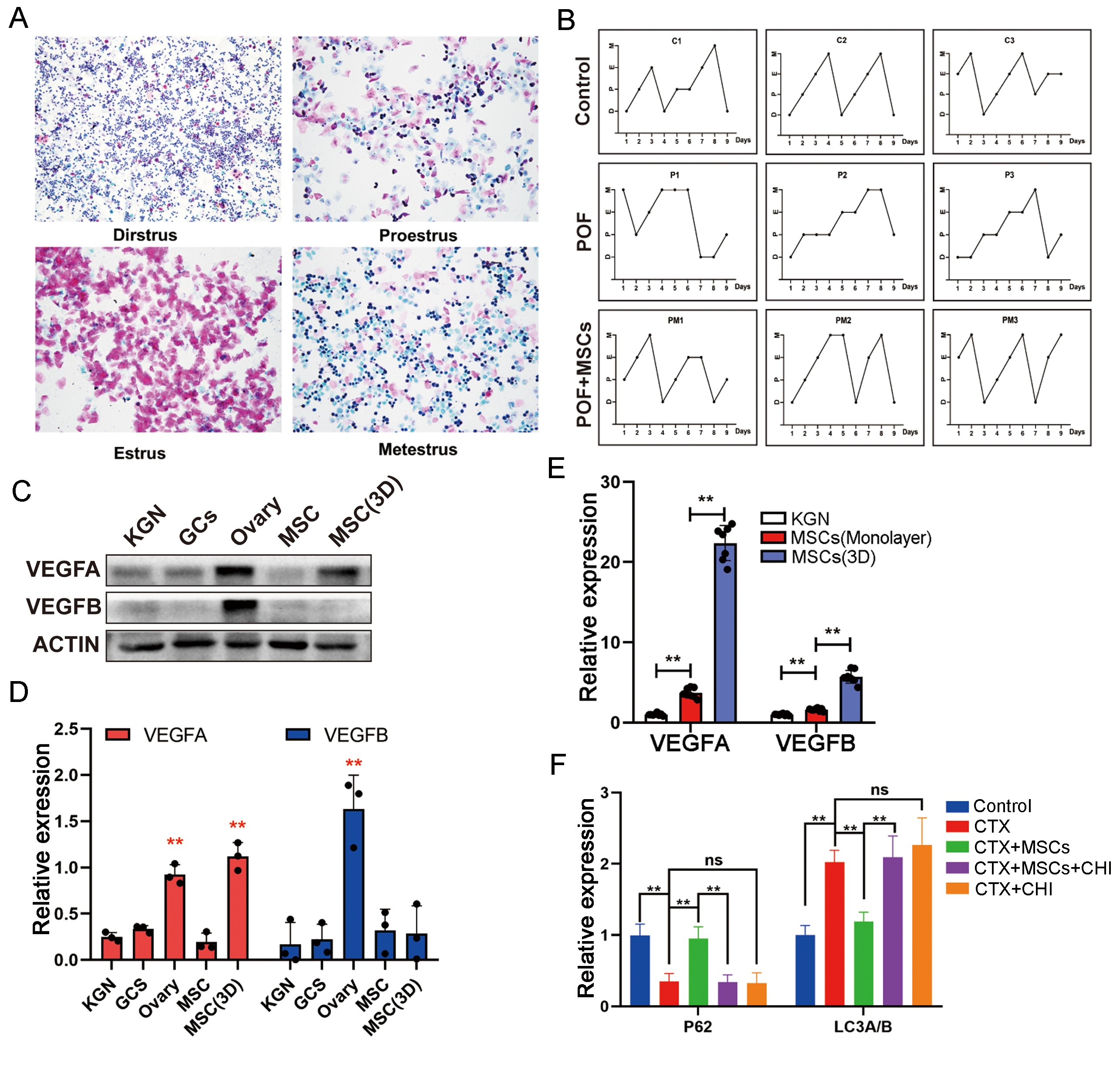


**Fig.S1 A** The morphology of vaginal cells during the four phases of the estrous cycle. **B** The phases distribution of the estrous cycle within a period of time. **C** The expression of VEGFA and VEGFB in ovary tissue and various cells (KGN, primary GCs, monolayer hUC-MSCs and 3D hUC-MSCs spheroid), and 3D hUC-MSCs spheroid could express more VEGFA than other cells. **D** Quantification of (**C**). **E** Comparison of VEGFA and VEGFB expression among KGN, monolayer hUC-MSCs and 3D hUC-MSCs spheroid in mRNA level. **F** Quantification of Fig. 6B.


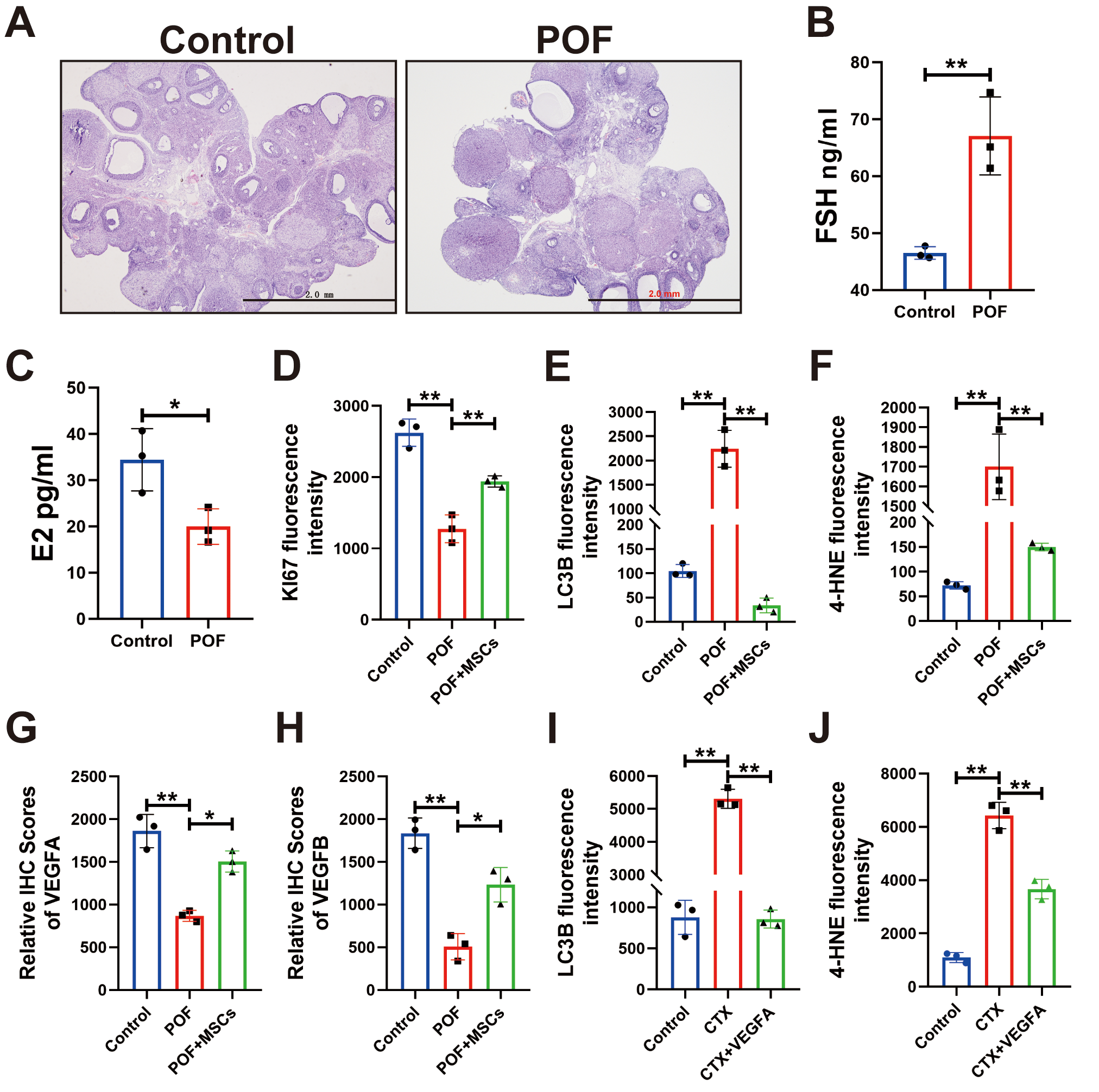


**Fig.S2 A** HE staining of animal ovarian tissue. The serum FSH (**B**) and E2 (**C**).

**D** Quantification of Fig. 2F. **E** Quantification of Fig. 3C. **F** Quantification of Fig. 3G. **G** Quantification of Fig. 4G. **H** Quantification of Fig. 4H. **I** Quantification of Fig. 8F. **J** Quantification of Fig. 8G.
